# Botanical, Traditional Use, Phytochemical, and Toxicological of *Arisaematis rhizoma*

**DOI:** 10.1155/2021/9055574

**Published:** 2021-11-30

**Authors:** Chun-yan Qi, Jing Wang, Xu Wu, Su-rong He, Qiao Zhang, Jian-hua Wu, Chong-bo Zhao

**Affiliations:** ^1^College of Pharmacy, Shaanxi University of Chinese Medicine, Xianyang 712046, China; ^2^College of Pharmacy, Engineering Technology Research Center of Shaanxi Administration of Chinese Herbal Pieces, Shaanxi University of Chinese Medicine, Xianyang 712046, China

## Abstract

**Materials and Methods:**

This review is a collection of all possible studies on AR, published in scientific journals, papers, and books. Using the papers related to *Arisaematis*, such as ScienceDirect, Wiley Online Library, Springer Link, Web of Science, CNKI, and WanFang Database. In this paper, the traditional uses, botany, phytochemistry, pharmacology, and toxicology of AR were reviewed. Finally, the existing problems and research directions of the research on AR are discussed.

**Results:**

Ninety-eight chemical constituents were isolated from AR. AR has a wide range of pharmacological effects, such as the effects on the central nervous system and cardiovascular system. It also has anti-tumor, sedative, analgesic, anticonvulsant, anti-inflammatory, expectorant, antiarrhythmic, anticoagulant, and other effects. It is also considered an effective drug for in vitro and in vivo validation.

**Conclusions:**

AR is an excellent traditional medicinal plant in China. Pharmacological studies support the traditional use of AR and may verify the folk use of AR in the treatment of different diseases. The anti-tumor effect of AR has been widely concerned by scholars at home and abroad. It has become a hot spot in recent years and has made great contributions to the survival and development of human beings. Although it has a high value of comprehensive utilization, its development and utilization are far from enough. Therefore, the comprehensive development of AR is worthy of further analysis.

## 1. Introduction

*Arisaematis rhizoma* (hereafter referred to as AR) is a tuber of the *Arisaema* plant of the *Araceae* family [1]. There are about 115 genera and more than 2,000 species in the *Araceae* family that are widely distributed all over the world, with more than 92% of them produced in the tropics. Among them, there are more than 90 species in the provinces of North and South China, 59 of which are endemic to China [2]. AR was first recorded as a herbal medicine in Shennong materia classic [3]. The tuber of *Pinellia pedatisecta* Schott was used as AR until the Song Dynasty (Kaibao Materia Medica) [4]. In the current market circulation, *Pinellia palmata* was widely used as a substitute for AR. The rhizomes of *Arisaema erubescens* (Wall.) Schott, *Arisaema amuren*se Maxim, and *Arisaema heterophyllum* Blume are all used as AR [5]. Because of its toxicity, AR was listed as one of the 28 toxic traditional Chinese medicine in clinical. If it is intended for oral administration, the processed AR (*Arisaematis rhizome* preparatum and *Arisaema cum* Bile) were preparation must be performed by an experienced individual. Previous works showed that AR contains lectins, alkaloids, flavonoids, steroids, terpenes, glycosides, amino acids, lignin, phenylpropanoids, and polysaccharides. In addition, many studies have found that AR has a wide range of pharmacological activities, including anti-tumor, anti-convulsant, anti-inflammatory, anti-bacterial, anti-arrhythmic, sedative and analgesic, anti-coagulant, and killing properties against the *Oncomelania* snail. Besides, AR is commonly recognized as a poisonous plant, and previous investigations have reported that the needle-like calcium oxalate crystals and lectins are the major toxic components of this plant [6].

This review is a collection of all possible studies on *Arisaema*, published in scientific journals, papers, and books. Using the papers related to *Arisaematis*, such as ScienceDirect, Wiley Online Library, Springer Link, Web of Science, CNKI, and WanFang Database. In this paper, the traditional uses, botany, phytochemistry, pharmacology, and toxicology of *Arisaema* were reviewed. Finally, the existing problems and research directions of the research on *Arisaematis* are discussed.

## 2. Traditional Usages and Modern Indications

AR has more than 2,000 years of history in China as a traditional medicinal plant [7]. It was found that AR had the functions of drying dampness and resolving phlegm, dispelling wind and calming nerves, and eliminating swelling and resolving knots. It can be used to treat apoplectic phlegm, mouth and eyes askew, hemiplegia, wind phlegm vertigo, epilepsy, convulsion, tetanus, scrofula, carbuncle, fall, and fold injury. Its external use can be used to treat carbuncle, snakebite, and other diseases. “*The classic of Materia Medica*” [8] record: “AR *is mainly used for wind phlegm*.” According to the compendium of “*Materia Medica in the Ming Dynasty*” [9], AR in the sky are spicy and numb, so they can cure wind and disperse blood; the temperature is dry, so they can overcome dampness and remove saliva; the nature is tight and poisonous, so they can attack accumulation and remove swelling, so they can cure tongue erosion.” At the same time, the toxicity of AR was indicated as “the AR is poisonous” in “*the classic of Materia Medica*” and “the *Chongyuan of Materia Medica*” [10]. Therefore, in clinical use, a unique Chinese processing method was created processing AR with alum and ginger in which the toxicity of AR decreases, while the effect of drying dampness and resolving phlegm increases.

At present, AR has become an important traditional Chinese medicine, which is commonly used in the treatment of tumors, rheumatoid arthritis, coronary heart disease, epilepsy, and other diseases [11]. At the same time, in order to better meet the clinical needs, the formulation of powder, pill, tablet, and other forms has been formed (Table 1).

In conclusion, most of the traditional clinical functions of AR are still currently being used.

1. “*Eliminating dampness and resolving phlegm*”: for treatment of accumulation of phlegm dampness, cough with abundant expectoration, inappetence, and oppressive chest.
2. “*Dispelling wind and stopping spasm*”: for treatment of dizziness due to wind phlegm, stroke with abundant phlegm, hemiplegia, and so on.
3. “*Detumescence*”: the fresh herb is used externally; it can subside swelling to cure furuncle and carbuncle with septicemia.

## 3. Botanical Aspects

**Figure 1:**
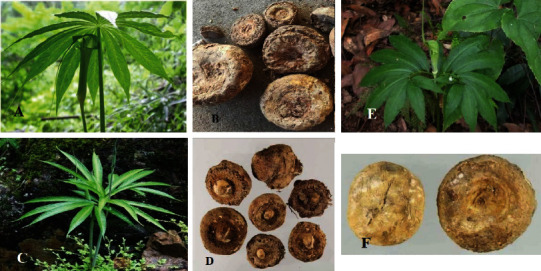
Photograph of *Arisaematis, Arisaema erubescens* (Wall.) Schott (a, b), *Arisaema amurense* Maxim. (c, d), and *Arisaema heterophyllum* Blume (e, f).

Three plants of AR consist of *A. erubescens* (Wall.) Schott., *Arisaema amuren*se Maxim., and *A. heterophyllum* Blume; all of them are perennial herbs of about 40–90 cm high. Its petiole is vertical and cylindrical and is about 40–55 cm long with a sheathed lower part. The base is covered with a transparent long membrane sheath and white green or purple spots on the long membrane sheath are present. The tuber is oblate with a straight diameter of about 2.5 to 5.5 cm. The outer skin of the tuber is yellow brown. The leaves are all split into leaflets, which are similar to palmate compound leaves, with 7–23 lobes that are lanceolate or long lanceolate in shape. This is about 13 to 19 cm in length and 1.5 to 2.5 cm in width. From the apex to the end, they are awn-shaped, whole, narrow, and wedge-shaped at the base, and the veins are feathery. Both sides of the leaves are smooth and glabrous, green above, and light green below (Figure 1) [12]. The flowers are dioecious, with a thick inflorescence axis, fleshy spikes, and club-like appendages at the apex. The peduncle is about 30 to 70 cm long, and the spathes are mostly green with a few purple ones. The male flowers have many stamens, with 2–4 clustered into a cluster; the anthers are black purple; and the female flowers are dense. Each flower is composed of one pistil; the ovary is oval; and the style is short. The berries of the plant are red. The flowering period of AR is from May to June, and the fruit period is August.

The height of *A. amurense* Maxim. (东北天南星) is about 35–60 cm (see Figure 1(c)), and the fibrous roots above it are distributed radially. The leaves are totally divided in the shape of bird toes, with five lobes (but only three lobes in the annual *Arisaema amurensis*), and they are obovate or broadly obovate, with a length of 11–15 cm and a width of 6–8 cm, all of which have irregular teeth. The inflorescence is 20–40 cm long, and the spathes are green or purple, with a total length of 11–14 cm. The inflorescence has rod-shaped appendages at the top. It is in berry red, flowers from July to August, and grows in a shady and humid forest on a shady slope.

*A. Heterophyllum* Blume (异叶天南星) is a perennial herb (see Figure 1(e)), with a height of 15–30 cm. There is a leaf 2–4 cm in diameter, with 13–19 lobes in bird's toe shape, a narrow tip and tapered base over the whole, lateral lobes 7–24 cm long and 2–6 cm wide, and the smallest central lobe. The flower stalk is extracted from the leaf sheath and is 30–55 cm long. The spathes is green; its lower part is tubular; and its upper part is bent downward like a helmet. With the inflorescence shape like a meat spike, the unisexual inflorescence is in the lower part of the male flower. The lower part of the bisexual inflorescence is a female flower, the upper part is a sparsely male flower, and the appendage at the top of inflorescence axis is mouse-tail-shaped, protruding. Berries are red when ripe. The flowering period is from April to May, and the fruit period is from July to September.

The dried tubers of AR are oblate and massive. The diameter is about 2 to 7 cm, and the thickness is about 1 to 2 cm. The surface is wrinkled or smooth, milky white or brown, with pitted fibrous root marks around (Figure 1(b)). A hard texture that is not easy to break, uneven section, white color, and powdered are among its characteristics. It has also pungent and spicy characteristics [1]. It is not suitable to take it as a medicine without skin removal.

The dried tubers of A. *amurense* Maxim. (东北天南星) are oblate (see Figure 1(d)), with a diameter of 1.5–4 cm. The tubers are marked with large and flat marks and prominent lateral buds.

The dried tubers of *A. heterophyllum* Blume (异叶天南星) are oblate and spherical (see Figure 1(f)), with a diameter of 1.5–4 cm, with concave marks on the tubers and a circle of obvious root marks around the tubers.

These plants grow in the wet forest on the shady slope. It is distributed in Hebei Province, Henan Province, Guangxi Province, Shaanxi Province, Hubei Province, Sichuan Province, Guizhou Province, Yunnan Province, Shaanxi Province, and other places in China.

## 4. Chemical Composition

The chemical composition of AR is complex, with more than 200 chemical compounds, including alkaloids, polysaccharides, lectins, amino acids, fatty acids, sterols, and lectins. In this paper, the chemical composition and the corresponding structure of AR are described in detail to provide some references for the in-depth study of the plant and provide ideas for its future research direction. It was reported that total alkaloids from AR could inhibit the proliferation and induce apoptosis of SF21 cells in a dose- and time-dependent manner, suggesting that total alkaloids from AR could induce programmed death of SF21 cells [13]. The pharmacodynamics study of total flavonoids and their main component, liphotropic side, showed that it had the effects of anti-ischemia, protecting nerve cells and analgesia [14]. Saponins are irritating to the gastric mucosa, which can reflexively increase the secretion of the trachea and bronchus and play the role of expectorant [15].

### 4.1. Glycosides

**Figure 2:**
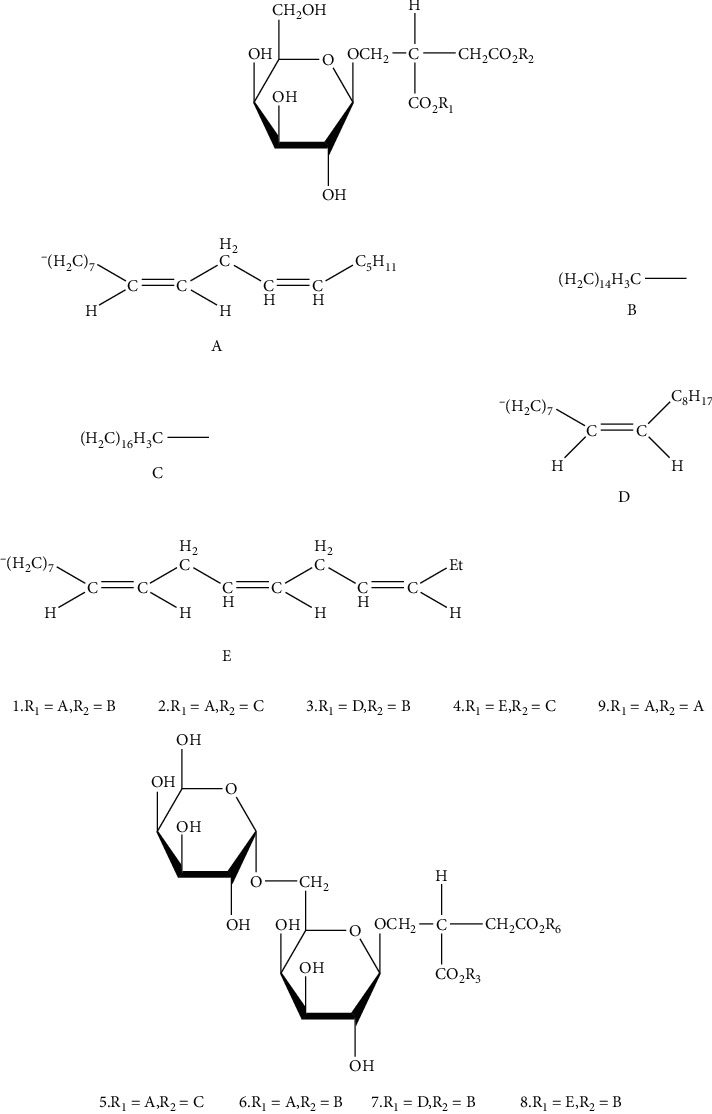
Chemical structure of *Arisaematis*.

**Figure 3:**
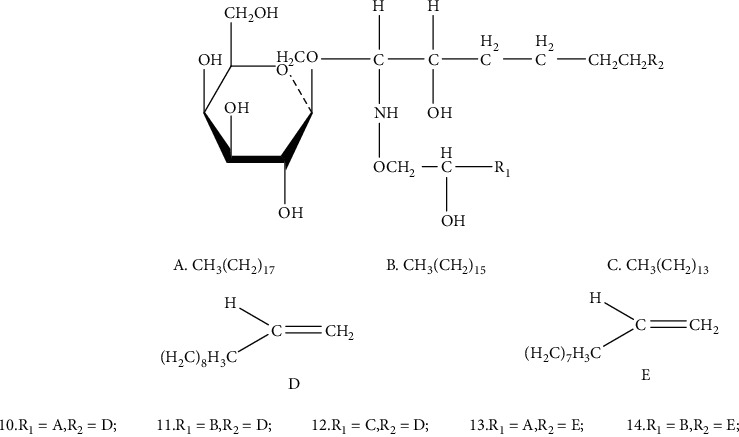
Chemical structure of diacylglycerol galactoside from *Arisaematis*: (a) CH_3_(CH_2_)_17_, (b) CH_3_(CH_2_)_15_, and (c) CH_3_(CH_2_)_13_.

Glycosides, also known as gametophytes, are compounds formed by the linkage of a sugar or a derivative of a sugar with another nonsugar substance via an end group carbon atom. At present, the main glycosides isolated from *Arisaematis* are oxyglycosides. Nine diacylglyceryl galactosides [16] (Table 2 and Figure 2) and five cerebrosides [17] (Table 3 and Figure 3) were isolated from AR. In addition, cerebrosides have the activity of protecting the liver [18].

### 4.2. Alkaloids

**Figure 4:**
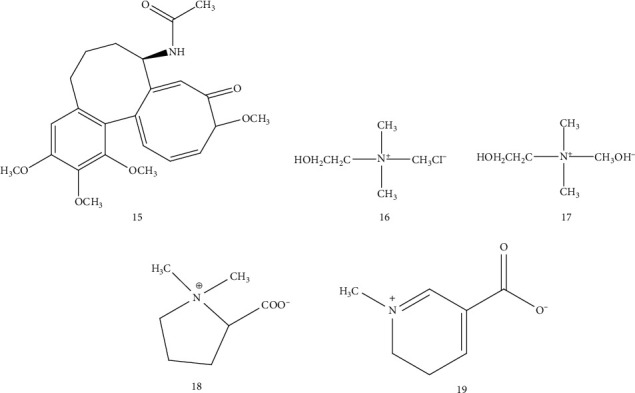
Chemical structures of alkaloids from *Arisaematis*.

The alkaloids have many important biological functions and physiological activities, which are abundant in the tuber of *Arisaematis*. The main alkaloids isolated from *Arisaematis* are trigonelline [19, 20], colchicine [21], choline chloride [22], choline, and stachydrine [16, 17, 19, 20] (Table 4 and Figure 4).

### 4.3. Fatty Acids and Sterols

**Figure 5:**
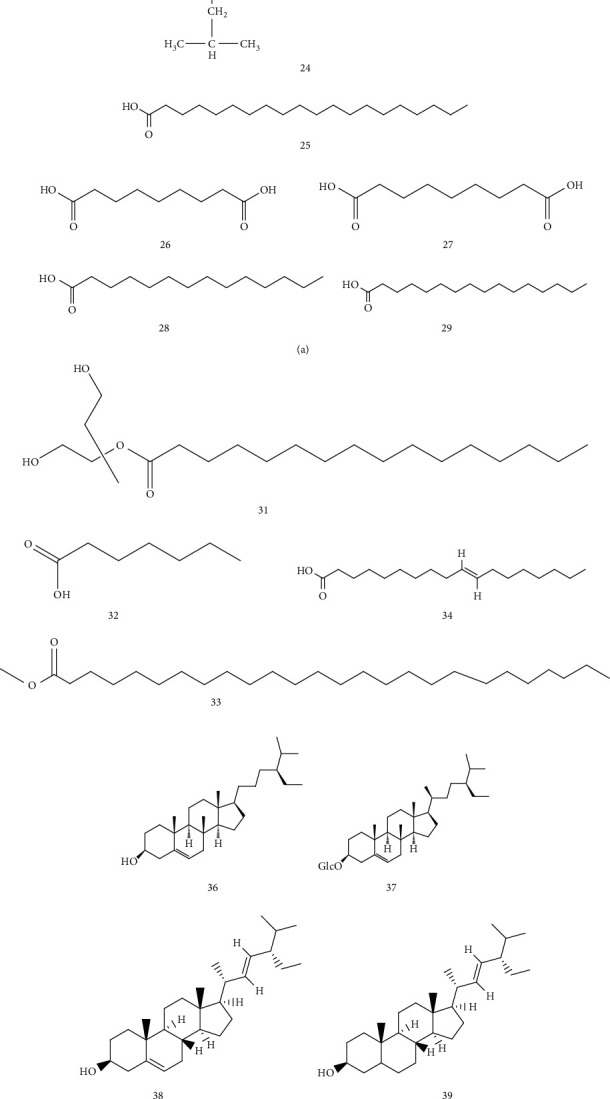
Chemical structures of fatty acids and sterols from *Arisaematis*.

Li Xuwen identified 16 fatty acids in the roots of AR by gas chromatography–mass spectrometry (GC-MS) [24] (Table 5 and Figure 5). Among them, the saturated fatty acid is the main component, while unsaturated fatty acid also has a certain proportion, accounting for 20% of the total fatty acid content. The main components include stearic acid, palmitic acid, and linoleic acid [25, 26]. Du Shushan obtained seven compounds from the petroleum ether fraction of the ethanol extract of AR [26]: triacontanoic aci, *β*-sitosterol [27], ethyl gallate, tetradecane, daucosterol [27–29], gallic acid, and hexadecanoic acid [30] (Table 6 and Figure 5).

### 4.4. Flavonoid

**Figure 6:**
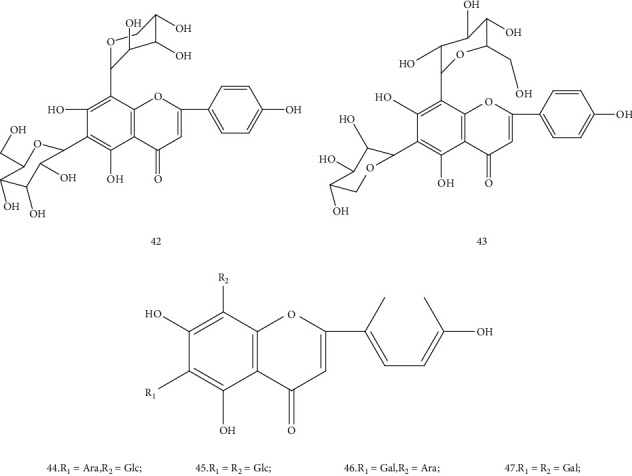
Chemical structure of flavonoids from *Arisaematis*.

Du Shushan isolated and identified six flavonoids from the tubers of AR, and all of them were flavonoid glycosides [27, 31]. Wang Guangshu also obtained six compounds from the ethanol extract of AR by macroporous resin, silica gel, and ODS column chromatography and were identified as flavonoids by physicochemical identification and spectral analysis [32] (Table 7 and Figure 6).

### 4.5. Lectin

Lectin is a type of protein or glycoprotein that can agglutinate red blood cells (RBCs). A variety of lectins have been found in AR, and blood agglutinin, lymph agglutinin, and sperm agglutinin have been found through cell research of humans and animals [33]. Wu Hao isolated a lectin AEL from *A. erubescens*, which has strong proinflammatory toxicity [34, 35]. Feng Li Xing isolated the lectin AHA from AR heterophylla that can induce autophagy of lung cancer A549 cells [36].

### 4.6. Amino Acids

**Figure 7:**
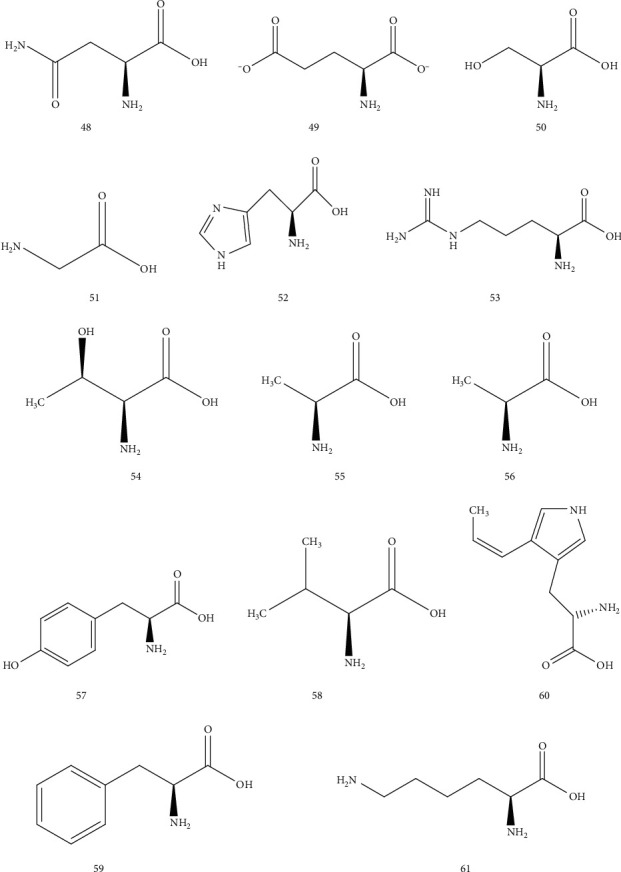
Chemical structure of amino acids in *Arisaematis*.

It is rich in amino acids in AR. Wang Xing found 14 kinds of amino acids were obtained from the qualitative and quantitative analysis of AR [37] (Table 8 and Figure 7).

### 4.7. Nucleosides

**Figure 8:**
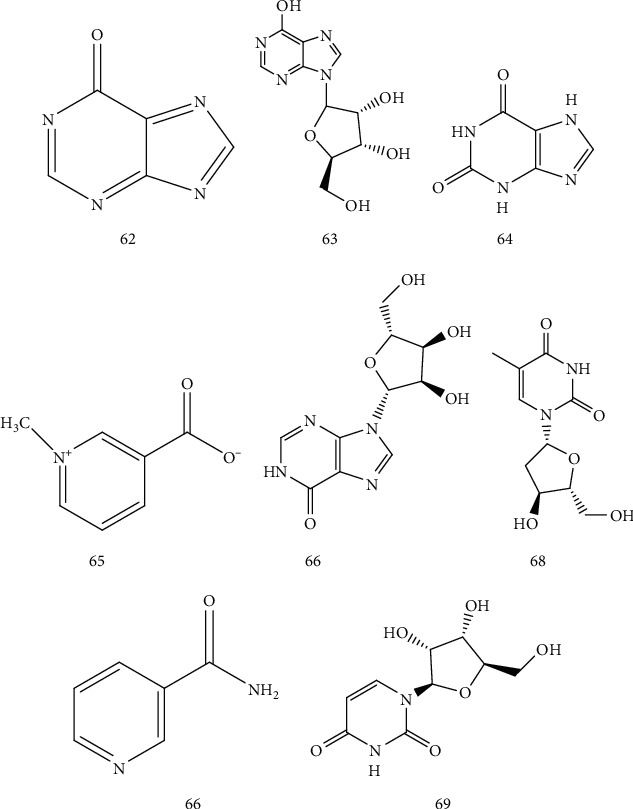
Chemical structure of nucleosides from *Arisaematis*.

Yi Jinghai eight nucleoside compounds were found in AR [38] (Table 9 and Figure 8). Nucleoside components have a wide range of physiological activities, are the basic components of biological cells to maintain life activities, and participate in the DNA metabolism process, with anti-tumor, anti-viral, immune regulation, treatment of cardiovascular and cerebrovascular diseases, and other functions [39].

### 4.8. Other Chemical Components

**Figure 9:**
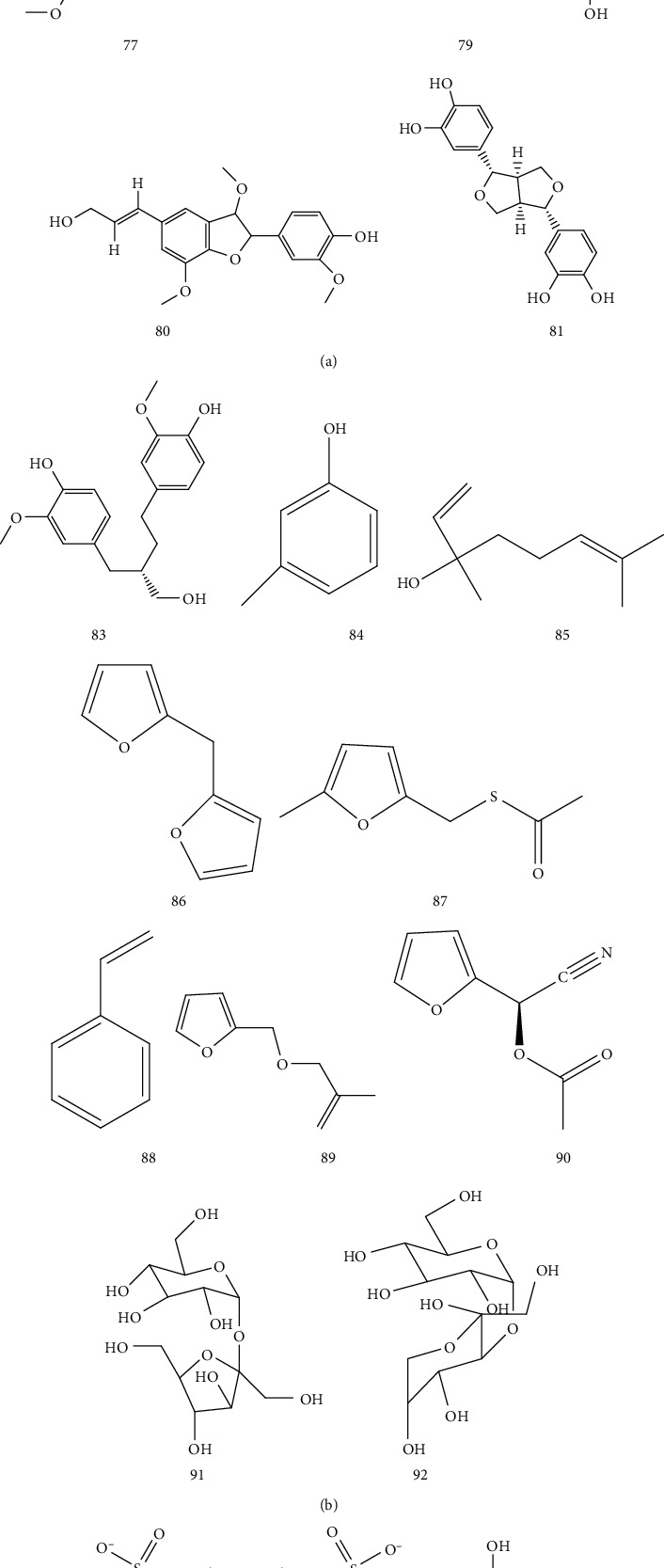
Other chemical structures of *Arisaematis*.

In addition, Fu Ming obtained 8 compounds from 80% ethanol extract of rhizome of AR [40] (Table 10 and Figure 9). Zheng Xiumei obtained 10 compounds from 80% ethanol extract of AR [41] (Table 10 and Figure 9); Yang Jia identified 52 compounds from AR by GC-MS, of which 7 compounds were more than 2% [42]. Sucrose and pine disaccharide were also isolated from AR [36] (Table 10 and Figure 9). In addition, there is a water-soluble polysaccharide in AR [43]; the monosaccharide composition of AR polysaccharide is mannose, rhamnose, glucose, galactose, arabinose, and fucose [6, 43, 44].

## 5. Pharmacological Action

AR has been reported to have a wide range of pharmacological activities, including the effects on the central nervous system and cardiovascular system; it is also used in the clinic for anti-tumor, sedation, analgesia, anti-convulsant, anti-inflammatory, expectorant, anti-arrhythmic, and anti-coagulant. In this section, we mainly introduce and analyze the pharmacological effects of AR (Table 10).

### 5.1. Effects on the Respiratory System

AR is a common traditional Chinese medicine for reducing phlegm. It has the effect of dryness and dampness reducing phlegm. The experiment was carried out by phenol red excretion method in mice showed AR aqueous solution (19 g/kg) had an expectorant effect [45]. Nie Rongzhen studied the anti-tussive and expectorant effects of AR by ammonia-induced cough test and phenol red excretion experiment in the trachea. The results showed that the cough frequency of mice could be significantly reduced, and the cough latency of mice could be prolonged by AR (4 g/kg) [46].

### 5.2. Effects on the Central Nervous System

#### 5.2.1. Anti-Convulsant Effect Activity (抗惊厥)

In recent years, experiments have shown that AR has a certain anti-convulsant effect. Zhao Naicai studied the anti-convulsant effect of the aqueous extract of AR (1 mL/20 g) on mice by subcutaneous injection of strychnine, caffeine, and pentamethylenetetrazol, indicating that the aqueous extract of AR has a certain anti-convulsant effect [47]. Oral administration of 60% ethanol extract (10.5 g crude drug/kg) of AR can antagonize tetrazole convulsion [48]. Chen Xiaoying studied the anti-convulsant effect of AR at different temperatures (30°C, 70°C, and 100°C) and found that intraperitoneal injection of a cold extract of *Arisaema* (30°C10 g/kg) could significantly inhibit the convulsion rate of strychnine and reduce the convulsion mortality [49]. It is also reported that intraperitoneal injection of AR decoction can increase the electric convulsion threshold of rabbits [50]. Liu Yuxi measured the anti-convulsant threshold and anti-convulsant effect of AR by a convulsion analyzer. The extract was injected intraperitoneally with 10 ml/kg (equivalent to 1.2 g/kg of crude drug). After administration of AR, the effect increased (119 ± 22 *μ*A), and the effect lasted for 7 days [51]. Li Xianduan investigated the synergism of different bile processing methods of AR with different bile processing methods by means of mice autonomous activity, sleep time, and convulsion test induced by pentylenetetrazol. The results showed that AR and its processed products could reduce the convulsion rate caused by pentylenetetrazol, reduce the number of spontaneous activities of mice, and prolong the sleep time of mice [52]. Wang Mingzheng concluded that the supercritical CO_2_ ethanol extract of AR could antagonize the maximal electric shock convulsion and pentylenetetrazole convulsion in a dose-dependent manner [53]. Nie Rongzhen used the mouse autonomous activity test and mouse drug convulsion experiment to conclude that AR has sedative and anti-convulsant effects and that AR can prolong the incubation period of convulsion induced by nicothamide [46].

#### 5.2.2. Sedative Effect Activity (镇静)

Intraperitoneal injection of AR decoction (10.5 g/kg) into rats and rabbits can prolong the sleep time of barbital sodium in mice, indicating that AR has an obvious sedative effect [48]. In addition, some studies have reported that the compound Sansheng needle of AR has an obvious sedative effect and has a significant sedative effect on pentobarbital [54]. Nie Rongzhen and others reported that AR can significantly reduce the spontaneous activity times of mice [46]. In addition, studies have reported that the water extract of *Arisaemw cum* Bile can prolong the sleep time induced by pentobarbital and significantly improve the sedative effect, which is manifested as the inhibition of walking distance, jumping, and vertical entry [55].

### 5.3. Anti-Inflammatory Effect Activity (抗炎)

The research shows that AR (the major component of compound Sansheng needle) is also a good anti-inflammatory drug. It can promote the exudation and absorption of inflammatory substances, inhibit tissue edema, and effectively reduce the permeability of capillaries [54]. Li Yang observed the anti-inflammatory effect of the extract of AR on different inflammatory models by using three models: xylene-induced auricle swelling, cotton-ball-induced granuloma proliferation, and acetic-acid-induced peritoneal capillary permeability increase in mice. The results showed that the alcohol extract and ethyl acetate extract of AR could significantly inhibit the auricle swelling induced by xylene, inhibit the proliferation of cotton ball granuloma in mice (external coating), and significantly inhibit the capillary permeability of mice induced by glacial acetic acid [56]. In addition, Liu Yanping found that high-dose preparation of AR could treat knee osteoarthritis in rabbits, reduce the content of IL-1 in synovial fluid, and reduce synovial inflammation. There was a positive correlation between the efficacy and the dose [57]. In addition, Zhao Chongbo used a type II collagen-induced arthritis rat model to study the anti-arthritis effect of water extract from AR and found that AR can be used for clinical treatment or prevention of rheumatoid arthritis [58]. In addition, it has been reported that AEL has proinflammatory activity, which may release inflammatory mediators through macrophages and induce foot swelling and neutrophil migration in rats. These findings suggest that AEL can be used as a tool to better understand the related mechanisms of the inflammatory response [59].

### 5.4. Analgesic Effect Activity (镇痛)

Xing Shulin has proved that AR (the major component of compound Sansheng needle) has an obvious analgesic effect [46]. Wang Qin studied the analgesic effect of AR flavonoids on Walker256 bone cancer pain rats. The results showed that AR flavonoids had a certain analgesic effect [60]. In addition, Nie Rongzhen used the hot plate method and acetic acid writhing test to observe the analgesic effect of AR. The results showed that AR could significantly reduce the number of writhing reactions induced by acetic acid in mice, playing an analgesic role [46]. Liu Chun compared the correlation between analgesia and toxicity of AR by hot plate method, The results showed that the percentage of pain threshold increased by more than 85% after 0.5 h of administration, indicating that AR has a certain analgesic effect [15]. In addition, the effects of crude AR and its different preparations combined with ginger juice or bile (*Arisaemw cum* Bile) on ICR mice were also reported. The results showed that the water extract of *Arisaemw cum* Bile could significantly improve the analgesic effect of mice [55]. Flavonoids are the main analgesic components in AR, but the research on the analgesic mechanism of AR is not deep enough.

### 5.5. Inhibitory Effect on Tumor Cells Activity (抗肿瘤)

With the research on chemical constituents of AR in recent years, the study of its anti-tumor effect has become a hot spot in recent years. Zhang Zhilin found that 2.5 g/L and 1.25 g/L ethanol extracts of AR had significant effects on the proliferation of mouse spleen cells [61]. Li Lingyan used the water extract of AR to observe the inhibitory effect of the drug on the tumor of mice after inoculation with H_22_, indicating that the water extract of AR can significantly inhibit the growth of H_22_ transplanted tumor in mice [62]. Jiang Shuang found that all treatment groups could inhibit the growth of S180 tumor by intragastric administration of AR polysaccharide (high, medium, and low doses (2g kg^−1^ d^−1^, 1g kg^−1^ d^−1^, and 0.5g kg^−1^ d^−1^)], and the inhibition rate of high dose group was 33.3%. It was found that all the treatment groups could inhibit the growth of S_180_ tumor, and the inhibition rate of the high-dose group was 33.3% [63]. Yang Zonghui reported that 95% alcohol of AR had an obvious inhibitory effect on SMMC7221 cells of liver cancer. The results showed that the extracts of AR in different concentrations (2 mg/mL, 4 mg/mL, and 8 mg/mL) had different anti-tumor effects [64]. Zhao Chongbo found that the inhibitory rate of AR polysaccharide on human lung cancer A459 was 8.625 mg·ml^−1^ [65]. Liang Feng and Meng Xiansheng used the MTT method to determine the inhibitory rate of the extract of AR on the proliferation of human lung cancer A549 cells, and the results showed that the extract of AR had a significant inhibitory effect on the A549 cells with IC_50_ of 133.5 g/mL [66, 67]. In addition, studies have shown that the total flavonoids of AR can inhibit the proliferation and apoptosis of lung cancer A549 cells [68]. Qiu Limin studied the treatment of human breast cancer MDA-MB-231 cells with AR polysaccharide, cisplatin, and AR polysaccharide + cisplatin and confirmed that the proliferation of MDA-MB-231 cells could be significantly inhibited by the combination of AR polysaccharide and cisplatin [69]. Tang Huayong verified the inhibitory effect of AR polysaccharides on the proliferation of human renal cancer cell line GRC-1 [70]. Li Feng explored the effect of water extract of AR on gastric cancer cells of rats and showed that the proliferation rate of cancer tissue cells in gastric cancer rats decreased under the intervention of water extract of AR [71]. Qi Xiaoxiao found that at the dose of (100 g/L), the intragastric administration of AR could slow down the growth of the tumor, and the tumor inhibition rate was 34.7% [72]. Qian Jinmao reported that the tumor inhibition rates of high, medium, and low dose groups of AR extract were 35.50%, 40.40%, 25.50%, 35.70%, 40.60%, and 24.30% [73]. Dong Wei intervened with the mice uterine fibroma with fresh AR water extract, which showed that fresh AR water extract could inhibit mouse uterine fibroma [74]. Tang Jianhua and others reported that the alcohol extract of AR could significantly inhibit the proliferation of K562, BGC823, and HeLa cells, with IC_50_ of 0.24 mg/mL, 0.78 mg/mL, and 82.17 mg/mL [75–77].

### 5.6. Other Functions Activity (其它)

In addition to the pharmacological effects described above, AR also has some other pharmacological effects. Rats were given 60% ethanol extract of AR (1.4 g/kg), which could not only delay the occurrence time of arrhythmia but also shorten the duration of arrhythmia [48]. It was found that the needle crystal of calcium oxalate had a significant lethal effect on *Oncomelania hupensis* [78]. Sensitivity test against Gram-negative bacteria showed that the ethanol extract of AR had an obvious inhibitory effect on both Gram-positive bacteria and Gram-negative bacteria [79]. In addition, it has been reported that AR can also whiten through inhibiting the production of melanin by regulating autophagy. The extract of AR was used to treat the melanin production of B16-F1 cells, regulate TRP1 protein and tryptophan enzyme, and reduce the anti-melanin production of RA in treated B16-F1 cells [80]. In addition, AR through the experiment of the influence of the needle crystal of calcium oxalate on the activity of *Oncomelania hupensis* and the examination of the scanning electron microscope has a cough-relieving effect [46].

## 6. Toxicity

In China, AR is traditionally and commonly recognized as a toxic plant as other plants in *Araceae*. Thus, the toxicities of AR have been comprehensively recorded and investigated. The toxic or irritant components of *Araceae* plants may be produced by special biological metabolites contained in the plants. It may include calcium oxalate needle crystal and its surface glycoprotein, and some trace polysaccharides [6, 81]. The content of calcium oxalate in the poisonous needle crystal is 90.26% [82]. The calcium oxalate needle crystal acts through special mucous cells [83], resulting in strong irritation and corrosiveness to the skin, mucous membrane, muscle, and other local tissues [84]. The toxicity of AR was mainly manifested in strong irritation to the oral cavity, throat, skin, and mucous membrane [85, 86]. After oral administration, it can cause numbness; stimulate the mucous membrane of the throat and gastrointestinal tract; and cause vomiting, abdominal pain, diarrhea, and other gastrointestinal and urinary tract irritation symptoms; when used externally, it can stimulate mucous membrane and skin, cause redness, burning sensation, blister, and even ulceration, which can affect muscle tissue.

### 6.1. Acute Toxicity

Yang Zhonglin and Wu Lianying carried out an acute toxicity study and maximum tolerance test of AR [87, 88] (Table 11). Dong Wei carried out an acute toxicity study on an ethanol extract of AR and obtained that LD_50_ was 155.78 g/kg [89]. In addition, Wu Zijun found obvious acute toxicity in mice by intraperitoneal injection of crude AR with LD_50_ of 21.58 g/kg [90]. Tang Liying intraperitoneal injection of AR powder, water extract, needle crystal, and processed product powder to compare the difference of acute toxicity. The acute toxicity test table showed that the LD_50_ of the AR needle crystal group was 42.53 mg·kg^−1^, the LD_50_ of the raw AR powder group was 1062 mg·kg^−1^, and the LD_50_ of the processed product powder group was 2788 mg·kg−^1^ [91] (Table 12).

### 6.2. Irritant Toxicity

Yang Zhonglin reported the results of the rabbit eye irritation test showed that the stimulation of prepared AR was lower than that of raw AR [87]. Wu Hao reported that the calcium oxalate needle crystal in the AR showed strong irritation to rabbit eyes, and the stimulating effect increased with the increase of concentration [81]. Tang Liying compared the toxicity of crude products, processed products, and needle crystal of AR through rabbit eye irritation experiment, which showed that the crude drug powder group and needle crystal group showed mild and moderate irritation, while the control group had no irritation reaction. However, it does not show that needle crystal is the main material basis for the stimulation of AR, and the needle crystal structure is destroyed after processing, which can reduce the irritation [88].

## 7. Pharmacokinetic Studies

So far, the pharmacokinetic studies on the extracts and compounds of this plant are very few. The pharmacokinetic studies of AR were mainly focused on flavonoid compounds, including schaftoside and isoschaftoside. Luo Fen studied the pharmacokinetic characteristics of schaftoside and isoschaftoside. In rats, the data are as follows: AUC (0⟶t) = 143.378 ± 26.249 h·*μ*g^–1^·mL^–1^, AUC (0⟶∞) = 149.106 ± 26.249 h·*μ*g^–1^·mL^–1^, *t*_1/2_ = 2.300 ± 0.756 h, *C*_max_ = 33.259 ± 3.192 *μ*g/L, AUC (0⟶t) = 167.122 ± 95.766 h·*μ*g^–1^·mL^–1^, AUC (0⟶∞) = 181.974 ± 108.915 h·*μ*g^–1^·mL^–1^, *t*_1/2_ = 2.592 ± 1.175 h, and *C*_max_ = 36.635 ± 19.53 *μ*g/L. It can be seen from the blood concentration-time curve of the two flavonoids that the blood concentration of the two flavonoids rises obviously at 3 h, which may be related to the enterohepatic circulation, absorption, and distribution in vivo [92]. Further research is needed to support it.

## 8. Future Perspectives and Conclusions

To sum up, AR is a traditional Chinese medicine with a long history. It has more than 2,000 years of application history and continuous research of modern science. At present, many chemical constituents have been isolated and identified from this plant. There is no doubt that AR is an important traditional Chinese medicine, although it has certain toxicity. For this reason, in the past many years, many experts are constantly studying the south star and have made great contributions in many aspects. At the same time, in the process of continuous research, there are still some new problems and challenges, which need further research and exploration to meet the clinical needs.

First of all, as a traditional Chinese medicine, AR contains glycosides, sugars, flavonoids, alkaloids, saponins, and other chemical components with expectorant, anti-inflammatory, anti-convulsant, anti-tumor, and other pharmacological effects. There are many studies on it, and the anti-tumor effect of AR has also been widely concerned by scholars at home and abroad and has become a research hotspot in recent years. However, the material basis of other pharmacological effects is not clear, and how and what kind of effects some chemical components play are not clear. It is necessary to further study its monomer compounds and clarify the material basis through certain methods and experiments; AR has certain toxicity. Toxicity studies showed that most of them were irritant, and the long-term toxicity was not obvious. There are some problems in toxicity evaluation methods; for example, oral generated AR can stimulate the throat and make the throat smooth and hoarseness, and it is difficult to enlarge the throat so it is necessary to find a toxicity evaluation model to better evaluate the toxicity of AR. There are too few studies on the pharmacokinetics of AR, which should be paid more attention to *in vivo* and *in vitro* verification to improve the safety of the clinical medication. So that AR can be better used better for the guidance of clinical medication to lay a certain foundation.

In general, as a commonly used traditional Chinese medicine, AR still needs further study. This paper systematically and comprehensively introduces the research status of AR at home and abroad in recent years, including traditional application, phytochemistry, pharmacology, and toxicology. Although great progress has been made in its research, there are still some problems in all aspects. Therefore, we still need to make continuous efforts to develop and utilize AR.[93]

## Figures and Tables

**Table 1:** Traditional and clinical application of *Arisaematis*.

Preparation name	Main compositions	Formulation	Traditional and clinical usages	Ref.
San Sheng Yin	*Arisaema amurense* Maxim.	Decoction	It is mainly used to treat apoplexy, faintness, askew mouth and eyes, and hemiplegia.	Qi Xiao Liang Fang 《奇效良方》
	*Aucklandia lappa* Decne.			
	*Aconitum carmichaelii* Debx.			
	*Aconitum carmichaelii* Debx.			

Yu Zhen San	*Angelica dahurica* (Fisch. ex Hoffm.)	Decoction	Tetanus	Chinese pharmacopoeia, 2020 《中国药典》
	*Arisaema amurense* Maxim.			
	*Typhonium giganteum* Engl.			
	*Gastrodia elata* Bl.			
	*Notopterygium incisum* Ting ex H. T. Chang			
	*Saposhnikovia divaricata*			
	(Turcz.) Schischk			

Hua Tan Wan	*Pinellia ternata* (Thunb.) Breit.	Pills	To dissipate phlegm. Main phlegm dampness, food accumulation and internal obstruction, cough and asthma, chest and diaphragm distention, and phlegm retention.	Chinese pharmacopoeia, 2020 《中国药典》
	*Arisaema amurense* Maxim.			
	*Gleditsia sinensis* Lam.			
	*Zingiber officinale* Rosc.			
	*Citrus reticulata* Blanco (Qingpi)			
	*Citrus reticulata* Blanco (Chenpi)			
	*Perilla frutescens* (L.) Britt.			
	*Raphanus sativus* L.			
	*Prunus armeniaca* L			
	*Pueraria lobata* (Willd.) Ohwi			
	*Hordeum vulgare* L.			
	*Crataegus pinnatifida* Bge			
	*Cyperus rotundus* L.			

Yi Zi San	*Cinnabaris*	Powder	Infantile apoplexy, hand, and foot contracture	Sheng Hu Juan Ba Shi San 《圣惠》卷八十三方》
	*Cicadae Periostracum*			
	*Bombyx Batryticatus*			
	*Pinelliae Rhizoma*			
	*Arisaematis Rhizom*			

Zhui Feng San	*Aconitum carmichaelii* Debx.	Powder	Dispel wind evil, clear the head, benefit the pharynx and diaphragm, dissipate phlegm, and saliva. It can cure headache, dizziness, palpitation, fever, pain, nasal congestion, neck and back, or itchy skin.	Mi Chuan Zheng Zhi Yao Jue Lei Fang 《秘传证治要诀类方》
	*Saposhnikovia divaricata*			
	(Turcz.) Schischk			
	*Ligusticum chuanxiong* Hort.			
	*Bombyx mori* Linnaeus			
	*Schizonepeta tenuifolia* Briq.			
	*Glycyrrhiza uralensis* Fisch.			
	*Typhonium giganteum* Engl.			
	*Notopterygium incisum* Ting exH. T. Chang			
	*Angelica dahurica* (Fisch. ex			
	Hoffm.) Benth. et Hook. f			
	*Arisaema amurense* Maxim.			
	*Gastrodia elata* Bl.			
	*Boszvellia carterii* Birdw.			
	*Pheretima vulgaris* Chen.			
	*Aconitum kusnezoffii* Reichb.			
	*Commiphora myrrha* Engl.			

Huo Luo Dan	*Aconitum carmichaelii* Debx.	Pills	Spleen blood cold for a long time, stagnation of meridians and collaterals, flowing foot and hand, muscle and pulse contracture fist, or redness and swelling, hard walking, heavy waist and legs, and pain of foot lifting.	Tai Ping Hui Min He Ji Ju fang 《太平惠民和剂局方》卷一
	*Aconitum kusnezoffii* Reichb.			
	*Pheretima vulgaris* Chen.			
	*Arisaema amurense* Maxim.			
	*Boszvellia carterii* Birdw.			
	*Commiphora myrrha* Engl.			

Qing Qi Hua Tan Wan	*Citrus reticulata* Blanco	Pills	It has the effect of clearing away heat and resolving phlegm, regulating qi, and relieving cough, and it is mainly used to treat all kinds of phlegm fire syndrome and phlegm heat syndrome.	Chinese pharmacopoeia, 2020 《中国药典》
	*Corydalis bungeana* Turcz			
	*Citrus sinensis* Osbeck			
	*Scutellaria baicalensis* Georgi			
	*Trichosanthes kirilowill* Maxim.			
	*Poria cocos* (Schw.) Wolf.			
	*Pinellia ternata* (Thunb.) Breit.			

Ding Xian Wan	*Gastrodia elata* Bl.	Pills	Epileptic seizures	Yi Xue Xin Wu 《医学心悟》
	*Fritillaria cirrhosa* D. Don			
	*Pinellia ternata* (Thunb.) Breit.			
	*Poria cocos* (Schw.) Wolf			
	*Acorus tatarinowii* Schott			
	*Buthus martensii* Karsch			
	*Bombyx mori* Linnaeus			
	*Citrus reticulata* Blanco (Chenpi)			
	*Polygala tenuifolia* Willd.			
	*Salvia miltiorrhiza* Bge.			
	*Ophiopogon japonicus* (L. f)			
	Ker-gawl			

Niu Huang Bao Long Wan	*Bos taurus domesticus* Gmelin	Pills	It has the effect of clearing away heat and shock, dispelling wind, and resolving phlegm. It is used for the convulsion caused by the excess of wind and phlegm in children.	Chinese pharmacopoeia, 2020 《中国药典》
	*Bambusa textilis* McClure			
	*Poria cocos* (Schw.) Wolf			
	*Moschus berezovskii* Flerov. dry secretions in the sachet of mature male musk deer			
	*Buthus martensii* Karsch			
	*Bombyx mori* Linnaeus			

Hua Feng Dan	*Notopterygium inchum* Ting ex H. T. Chang	Pills	Expectorant, anti-pyretic, and convulsive. Treat children with convulsion and epilepsy.	Yin Tong Bai Wen Juan Shang 《婴童百问》卷上
	*Angelica pubescens* maxim. f. *biserrata* Shan et yuan			
	*Saposhnikovia divaricata*			
	(Turcz.) Schischk			
	*Gastrodia elata* Bl.			
	*Panax ginseng* C. A. Mey dried roots and rhizomes			
	*Ligusticum chuanxiong* Hort. dried roots			
	*Schizonepeta Tenuifolia* Briq. dried aboveground part			
	*Buthus martensii* karsch dried bulk			

Hu Po Bao Long Wan	*Dioscorea opposita* Thunb. dried roots	Pills	Clearing heat and phlegm, calming, and tranquilizing. It is used for the acute convulsion of phlegm food type caused by internal food injury. The symptoms include fever, convulsion, restlessness, phlegm panting, and convulsion.	Yin Tong Bai Wen Juan Liu 《婴童百问》卷六
	*Glycyrrhiza uralensis* Fisch. dried roots and rhizomes			
	*Bambusa textili*s McClure dry fluid			
	*Santalum album* L. dried heartwood			
	*Citrus aurantium* L. dried and immature fruit (Zhiqiao)			
	*Citrus aurantium* L. dried and immature small fruit (Zhishi)			
	Panax ginseng C.A. Mey			

Zhi Ke Hua Tan Wan	*Ephedra sinica* Stapf. dried grass stem	Pills	Relieving cough and phlegm, relieving asthma. It is used for asthma.	Chinese pharmacopoeia, 2020 《中国药典》
	*Belamcanda chinensis* (L.) DC dried roots			
	*Citrus reticulata* Blanco (Chenpi)			
	*Stemona japonica* (BL) Miq. dried root			
	*Tussilago farfara* L. dried flower bud			
	*Pinellia ternata* (Thunb.) Breit. dried tuber			
	Platycodon grandiflorum (Jacq.) A.DC dried root			
	*Schisandra chinensis* (Turcz.) Baill. dried fruit			
	*Zingiber officinale* Rosc. dried rhizome			
	*Glycyrrhiza uralensis* Fisch. dried roots and rhizomes			
	*Descurainia sophia* (L.) Webb. ex Prantl. dried seed			
	*Asarwm sieboldii* Miq. dried roots and rhizomes			
	*Ziziphus jujuba* Mill. dry ripe fruit			
	*Curcuma longa* L. dry root tubers			

Jin Su Dan	*Bombyx mori* Linnaeus	Pills	For children with wind phlegm convulsion.	Chinese pharmacopoeia, 2020 《中国药典》
	*Buthus martensii* Karsch. a dried worm			
	*Typhonium giganteum* Engl. dried tubers			
	*Moschus berezovskii* Flerov. dry secretions in the male sachet			
	*Gastrodia elata* Bl. dried tubers			
	*Boszvellia carterii* Birdw. dried resin			

Xiao Er Zhi Bao Wan	*Perilla frutescens* (L.) Britt. dried leaves	Pills	Phlegm leading stagnation. It is used for children with cold, fever and stuffy nose, cough and phlegm, and vomiting and diarrhea.	Chinese pharmacopoei, 2020 《中国药典》
	*Pogostemon cablin* (Blanco) Benth. dry above-ground parts			
	*Mentha haplocalyx* Briq. dry above-ground parts			
	*Notopterygium inchum* Ting ex H. T. Chang dried roots and rhizomes			
	*Citrus reticulata* Blanco (Chenpi)			
	*Typhonium giganteum* Engl. dried tubers			
	*Sinapis alba* L. dried seed			
	*Fritillaria cirrhosa* D. don dried bulbs			
	*Areca catechu* L. dried seed			
	*Crataegus pinnatifida* Bge.			
	*Poria cocos* (Schw.) Wolf.			
	*Hordeum vulgare* L.			
	*Gastrodia elata* Bl. dried tubers			
	*Uncaria macrophylla* wall stem branches with dry hooks			
	*Bombyx mori* Linnaeus			
	*Cryptotympana pustulata* Fabricius the shell of a larva that falls off during eclosion			
	*Buthus martensii* Karsch. a dried worm			

Xiao Er Kang Xian Jiao Nang	*Arisaema cum* Bile, *Pinelliae rhizoma*	Capsule	It can eliminate phlegm and wind, strengthen the spleen, and regulate qi. It is used for the syndrome of wind phlegm blocking in children with primary generalized tonic clonic seizure. When the seizure occurs, there are convulsions of limbs, salivation at the mouth, straggling of eyes, and even fainting.	Chinese pharmacopoeia, 2020 《中国药典》
	*Gastrodiae rhizoma, Pseudostellariae radix, Poria, Citri exocarpium rubrum*			
	*Rhizoma anemones Altiaicae, Canarii fructus, Ambrum, Aquilariae lignum resinatum, Massa medicata fermentata, Aurantii fructus, Chuanxiong rhizoma, Notopterygii rhizoma Et radix*			

**Table 2:** Diacylglyceryl isolated from *Arisaematis*.

No.	Name	Ref.
1	(2S)-1-0-hexadecyl-2-O(9Z,12Z-octadecenyl)-3-O-*β*-d-galactopyranose glycerol	[17]
2	1-O-(9Z-octadecyl)-2-0-(9Z,12Z-octadecenyl)-3-0-d-galactopyranosylglycerol	[17]
3	1-0-hexadecyl-2-0-(9Z-octadecenyl)-3-O-*β*-d-galactopyranose glycerol	[17]
4	1-0-octadecyl-2-0-(9Z,12Z,5Z-octatrienyl)-3-O-*β*-d-galactopyranosylglycerol	[17]
5	(2S)1-0-octadecyl-2-0-(9Z,12Z-octadienyl)-3-O-[*α*-D-galactopyranosyl-(1″⟶6′)O-*β*-D-galactopyranosyl]glycerol	[17]
6	1-O-hexadecyl-2-0-(9Z,12Z-octadecadienyl)-3-0-[*α*-D-galactopyranosyl-(1″⟶6′)-O-*β*-D-galactopyranosyl]glycerol	[17]
7	1-O-hexadecyl-2-O-(9Z-octadecenyl)-3-O[*α*-D-galactopyranosyl-(1″⟶6′)-O-*β* -D-galactopyranosyl] glycerol	[17]
8	1-O-hexadecyl-2-O-(9Z,12Z,15Z-octatrienyl)-3-0-[*α*-D-galactopyranosyl-((1″⟶6′)-O-*β*-D-galactosyl]glycerol	[17]
9	1-O-(9Z,12Z-octadecadienyl)-2-O-(9Z,12Z-octadienyl)-3-0-d-galactopyranosylglycerol	[17]

**Table 3:** Cerebrosides isolated from *Arisaematis*.

No.	Name	Ref.
10	1-O-*β*-D-glucopyranosyl-(2S,3R,4e,8Z)-2-[(2(R)-hydroxy eicosyl)phthalamido]-4,8-octadecene-1,3-diol	[16]
11	1-O-*β*-D-glucopyranosyl-(2S,3R,4e,8Z)-2-[(2(R)-hydroxyoctadecyl)acylamino]-4,8-octadecadien-1,3-diol	[16]
12	1-O-*β*-D-glucopyranosyl-(2S,3R,4e,8Z)-2-[(2(R)-hydroxyhexadecyl)amido]4,8-octadecene-1,3-diol	[16]
13	1-O-*β*-D-glucopyranosyl(-2S,3R,4e,8e)-2-[(2 (R)- hydroxy eicosyl)acylamino]4,8-octadecene-1,3-diol	[16]
14	1-O-*β*-d-pyranoglucosyl-(2S,3R,4e,8e)-2-[(2(R)-hydroxyoctadecyl)amide]-4,8-octadecene-1,3-diol	[16]

**Table 4:** Alkaloids isolated from *Arisaematis*.

No.	Name	Ref.
15	Colchicine	[19]
16	Cholinechlorid	[20]
17	Choline	[23]
18	Stachydrine	[23]
19	Cucurbitacin	[19,20]

**Table 5:** Fatty acid composition isolated from *Arisaematis*.

No.	Name	Ref.
20	Palmitic acid (25.21%)	[24]
21	Stearic acid (22.44%)	[24]
22	Linoleic acid (14.03%)	[24]
23	Linolenic acid (5.48%)	[24]
24	Hexadecyl heptadecanoic acid (5.11%)	[24]
25	Arachidic acid (4.49%)	[24]
26	Azelaic acid (3.05%)	[24]
27	Azelaic acid (2.51%)	[24]
28	Pentadecanoic acid (2.10%)	[24]
29	Heptadecanoic acid (1.97%)	[24]
30	Hexagoic acid- 2-isoproyl-2-methyl-5-oxo (1.72%)	[24]
31	Palmitic acid-3-hydroxy (1.20%)	[24]
32	Caprlylic acid (0.68%)	[24]
33	Tetralosanoic acid (0.63%)	[24]
34	Isooleic acid (0.49%)	[24]

**Table 6:** Sterols isolated from *Arisaematis*.

No.	Name	Ref.
35	Triacontanoic acid	[26]
36	*β* sitosterol	[27, 30]
37	Carotene	[28–30]
38	Stigmasterol	[30]
39	Sitosterol	[30]
40	Rapeseed sterol	[30]
41	Cholesterol	[30]

**Table 7:** Flavonoids isolated from *Arisaematis*.

No.	Name	Ref.
42	Schaftoside	[31, 32]
43	Isoschaftoside	[31, 32]
44	Apigenin-6-c-arabinogalactoside	[31, 32]
45	Apigenin-6,8-di-c-glucopyranoside	[31, 32]
46	Apigenin-6-c-galacto-8-c-arabinoside	[31, 32]
47	Apigenin-6,8-di-c-galactoside	[31, 32]

**Table 8:** Amino acids isolated from *Arisaematis*.

No.	Name	Ref.
48	Asparagine	[37]
49	Glutamate	[37]
50	Serine	[37]
51	Glycine	[37]
52	Histidine	[37]
53	Arginine	[37]
54	Threonine	[37]
55	Alanine	[37]
56	Proline	[37]
57	Tyrosine	[37]
58	Valine	[37]
59	Phenylalanine	[37]
60	Tryptophan	[37]
61	Lysine	[37]

**Table 9:** Nucleosides isolated from *Arisaematis*.

No.	Name	Ref.
62	Uracil	[38]
63	Hypoxanthine	[38]
64	Xanthine	[38]
65	Uridine	[38]
66	Inosine	[38]
67	Guanosine	[38]
68	Thymidine	[38]
69	Adenosine	[38]

**Table 10:** Other chemical compounds isolated from *Arisaematis*.

No.	Name	Ref.
70	Larch alcohol	[40]
71	Ferulic acid	[40]
72	P-hydroxycinnamic acid	[40]
73	P-hydroxybenzoic acid	[40]
74	Caffeic acid	[40]
75	Guaiacyl glycerol	[40]
76	Oleoresin	[40]
77	Larch resin	[41]
78	Pinoresinol	[41]
79	Isoamericanol A	[41]
80	Dehydrodiconiferyl alcohol	[41]
81	3,3′-bisdemethylpinoresinol	[41]
82	Americanol A	[41]
83	Larch resin	[41]
84	m-Cresol (5.31%)	[42]
85	Linalool L (3.69%)	[42]
86	Furan, 2, 2′ -Methylenebis (2.8%)	[42]
87	2-Furfuryl -5-Methylfuran (2.52%)	[42]
88	Styrene (2.48%)	[42]
89	Furan, 2 -(2-propenyl) (2.15%)	[42]
90	2-Furanmethanol, acetate (2.12%)	[42]
91	Sucrose	[42]
92	Disaccharide	[42]
93	Decosanoic acid (4.51%)	[30]
94	Ethyl gallate	[26]
95	Forty alkanes	[26]
96	Gallic acid	[26]
97	Hexadecanoic acid	[26]
98	Mannitol	[30]

**Table 11:** Toxicity study of *Arisaematis* and *Arisaematis Rhizoma Preparatum*.

Extract/compound	Animal	LD_50_/toxic dose range	Toxic effects	*In vitro/in vivo*	Ref.
*A. amurense* maxim.	Mice	0.4 mL/10g	Death	*In vivo*	[87]
AR	Mice	12 g/kg	60% death	*In vivo*	[88]
Processed AR	Mice	12 g/kg	Normal	*In vivo*	[88]
AR	Mice	LD_50_ = 155.78 g/kg^−1^	Death	*In vivo*	[89]
AR	Mice	LD_50_ = 21.508 g/kg^−1^	Death	*In vivo*	[90]
AR needle crystal	Mice	LD_50_ = 42.53 mg·kg^−1^	Death	*In vivo*	[91]
AR powder	Mice	LD_50_ = 1,062 mg·kg^−1^	The abdomen is swollen, and the fur is dull	*In vivo*	[91]
Processed AR powder	Mice	LD_50_ = 2,788 mg·kg^−1^	The abdomen is swollen, and the fur is dull	*In vivo*	[91]

**Table 12:** Pharmacological effects of *Arisaematis rhizoma*.

Pharmacological action	Details	Result	*In vitro/in vivo*	Ref.
Anti-tumor activity	The anti-tumor activity of ethanol extract of AR was determined by inoculating tumor cells in mouse armpit	10 g/L and 5 g/L groups had significant effects on the proliferation of mouse spleen cells	*In vitro*	[61]
	A mouse model of subcutaneous transplantation of H_22_ liver cancer was established	Intragastric administration of 100 g/L AR could slow down the growth of the tumor, and the inhibition rate was 34.7%	*In vivo*	[61]
	To observe the effect of *Arisaematis* extract on human hepatoma SMMC-7721 cells	4.8 mg/mL could induce SMMC-7721 cell apoptosis	*In vitro*	[63]
	To investigate the inhibitory effect of alcohol extract and water extract of AR on the growth of S_180_ sarcoma in mice	The anti-tumor rates of ethanol extract and water extract in high, medium, and low dose groups were 35.5%, 40.4%, 25.5%, 35.75%, 40.6%, and 24.3%	*In vitro*	[73]
	To observe the anti-tumor effect of polysaccharide from AR in vitro	IC_50_ = 8.625 mgmL^−1^	*In vitro*	[65]
	The effect of AR polysaccharides on the proliferation of human renal cell line GRC-1	Compared with the blank group, ARPS significantly inhibited the proliferation of GRC-1 cells at 20–200 mgL^−1^		[67]
	MTT method was used to determine the inhibitory rate of alcohol extract and water extract of AR on proliferation of human lung cancer A549 cells	IC_50_ _=_ 64.46 *μ*g/mL; IC_50_ _=_ 133.5 *μ*g/mL	*In vitro*	[71]
	To explore the effect of water extract of AR on the expression of PKM2 and mTOR in gastric cancer cells of rats	The levels of motilin and gastrin in the high concentration group were 137.65 pg/mL and 88.76 pg/mL, and the apoptosis rate was 49.73%	*In vitro*	[72]
	MTT method was used to determine the inhibitory effect of alcohol extract of AR on human K562 cells	The results showed that the ethanol extract could significantly inhibit the proliferation of human K562 cell line, IC_50_ = 65.07 *μ*g·mL^−1^	*In vitro*	[75]
	Human erythroleukemia cell line K562, human gastric cancer cell line BGC823, and human cervical cancer cell line HeLa were used to determine the inhibitory effect of alcohol extract and water extract of AR on tumor cells in vitro	The IC_50_ of ethanol extract was 65.07 *μ*g/mL, 0.59 mg/mL, and 5.11 mg/mL, respectively; the IC_50_ of water extract was 0.24 mg/mL, 0.78 mg/mL, and 82.17 mg/mL, respectively	*In vitro*	[76]
	To observe the anti-tumor effect of water extract of AR on transplanted tumor H_22_ in mice	The tumor inhibition rate of high dose group was 38.9%	*In vitro*	[47]

Anti-convulsant effect	The anti-convulsant effect of water extract of AR (1 mL/20g) was studied by subcutaneous injection of strychnine, caffeine, and pentamethylenetetrazol in mice	The water extract of AR has an anti-convulsant effect	*In vivo*	[48]
	To study the anti-convulsant effect of AR at different temperatures (30°C, 70°C, and 100°C)	The convulsion rate of strychnine was inhibited by intraperitoneal injection of a cold extract of AR (10 g/kg at 30°C)	*In vivo*	[50]
	The anti-convulsant effect of AR against pentylene tetra plastic convulsion was measured by a convulsion analyzer	After administration of AR, it increased 119 ± 22 *μ*A, and the effect lasted for 7 days	*In vivo*	[51]
	To investigate the effects of different bile processed AR on spontaneous activity, sleep time, and convulsion induced by pentylenetetrazol in mice	AR can reduce the convulsion rate caused by pentylenetetrazol	*In vivo*	[53]
	To study the anti-convulsant effect of supercritical CO_2_ ethanol extract from AR	Dose-dependent antagonism of maximal electric shock convulsion in mice	*In vivo*	[46]

Sedative effect	Rats and rabbits were intraperitoneally injected with AR decoction (crude drug of AR 10.5 g/kg)	Prolonging the sleeping time of barbital sodium in mice	*In vivo*	[49]
	The sedative effect was compared by spontaneous activity test in mice	The results showed that AR could reduce the spontaneous activity of mice	*In vivo*	[54]

Analgesic effect	The analgesic effect of AR was observed by hot plate test and acetic acid writhing test in mice	It can significantly reduce the number of writhing reactions induced by acetic acid in mice and play an analgesic role	*In vivo*	[54]
	To explore the effect of crude AR and processed products on ICR mice	The water extract of *Rhizoma* AR can obviously improve the analgesic effect in mice	*In vivo*	[60]
	Analgesic effect of AR flavonoids on Walker256 bone cancer pain in rats	Total flavonoids of Rhizoma Arisaematis nanogel may have an analgesic effect in the development of bone cancer	*In vivo*	[15]

Anti-inflammatory effect	To observe the anti-inflammatory effect of extract of AR on different inflammatory models	The extract of AR can obviously inhibit the auricle swelling of mice induced by xylene	*In vivo*	[56]
	To investigate the effect of AR on IL-1 and synovium of knee osteoarthritis in rabbits	A high dose of AR can reduce the content of IL-1 in synovial fluid and synovial inflammation in rabbits with knee osteoarthritis	*In vivo*	[58]

Other pharmacological effects	The experiment was carried out by phenol red excretion method in mice	The water extract of AR (19 g/kg) has an expectorant effect	*In vivo*	[48]
	The 60% ethanol extract of A. *parachinensis* and AR were observed	AR (1.4 g crude drug/kg) can delay arrhythmia	*In vivo*	[78]
	Treatment of *Oncomelania hupensis* with needle crystal of calcium oxalate in AR	The needle crystal of calcium oxalate has a lethal effect on *Oncomelania hupensis*	*In vitro*	[78]
	The sensitivity of alcohol extract of AR to Gram-negative bacteria was studied	The alcohol extract of *Rhizoma* AR had an inhibitory effect on Gram-positive and Gram-negative bacteria		[79]
	The melanin of B16-F1 cells was treated with the extract of AR	Schaftoside can inhibit the production of melanin and play a whitening role	*In vitro*	[80]
